# Radical total gastrectomy for gastric cancer complicated by hepatic sinusoidal obstruction syndrome: a case report

**DOI:** 10.3389/fmed.2025.1544400

**Published:** 2025-05-09

**Authors:** Bin Gao, Jingjing Zhang, Lei Zhu, Yaming Zhang

**Affiliations:** Department of General Surgery, Anqing Municipal Hospital, Anqing, China

**Keywords:** hepatic sinusoidal obstruction syndrome, gastric cancer, pyrrolizidine alkaloids, liver failure, HSOS

## Abstract

Hepatic sinusoidal obstruction syndrome (HSOS) is often caused by the ingestion of pyrrolizidine alkaloids (PAs). To date, research on PAs-induced HSOS remains limited. Due to differing etiologies of HSOS in Western and Eastern populations, the clinical features, imaging findings, treatment approaches, and outcomes of HSOS caused by hematopoietic stem cell transplantation or oxaliplatin may not be directly applicable to PAs-induced HSOS. PAs-induced HSOS commonly presents with painful hepatomegaly, ascites, and jaundice. Laboratory tests commonly show abnormal liver function in patients with PA-induced HSOS. Contrast-enhanced computed tomography and magnetic resonance imaging often reveal distinctive imaging features and significant histopathological liver changes in PAs-induced HSOS. These findings highlight the effectiveness of radiological imaging and liver biopsy as diagnostic tools. Treatment strategies for PAs-induced HSOS include fluid management, anticoagulation therapy, glucocorticoids, transjugular intrahepatic portosystemic shunt (TIPS), and liver transplantation. However, managing PAs-induced HSOS remains challenging. This paper presents the case of an elderly male diagnosed with gastric cancer complicated by hepatic sinusoidal obstruction syndrome. The diagnosis was based on characteristic imaging findings, a history of pyrrolizidine alkaloids ingestion, and standard diagnostic criteria, including liver biopsy and histological examination. The patient recovered fully after timely diagnosis and treatment, which included radical total gastrectomy, hepatoprotective diuretics, albumin supplementation, and low-molecular-weight heparin therapy.

## 1 Introduction

HSOS is a type of obliterative venulitis that affects terminal hepatic venules. The classic symptom triad includes weight gain, painful hepatomegaly, and jaundice. Diagnosis relies on medical history, clinical presentation, laboratory tests, imaging, biomarkers, and hepatic histopathology (1–3). In developed countries, HSOS is mainly caused by myeloablative therapy prior to hematopoietic stem cell transplantation or oxaliplatin-based chemotherapy (4–6). In China, HSOS is primarily caused by consuming herb or dietary supplements containing Pas (1, 7, 8). The differing etiologies of HSOS in Western and Chinese populations make the definitive diagnosis of PA-induced HSOS particularly challenging. Mild HSOS is often self-limiting, while severe cases can progress to multiple organ failure with high mortality (7, 9). The mortality rate of PA-induced HSOS ranges from 16% to 40%, with hepatic failure as the leading cause of death (10, 11). In severe cases, besides conventional treatments such as hepatoprotective therapy, albumin infusion, and diuretics, defibrotide and anticoagulation serve as primary medical therapies. The treatment aims to alleviate hepatic sinusoidal and small vein congestion, lower portal vein pressure, and enhance liver function (12, 13). However, managing PA-induced HSOS remains challenging for hepatologists. No specific treatment for PA toxicity has been established, particularly for critically ill patients. This article presents a rare case of HSOS induced by six months of Tu-San-Qi wine consumption, complicated by gastric cancer. After a comprehensive safety evaluation, the patient underwent curative-intent total gastrectomy and achieved remission.

## 2 Case report and treatment process

**FIGURE 1:**
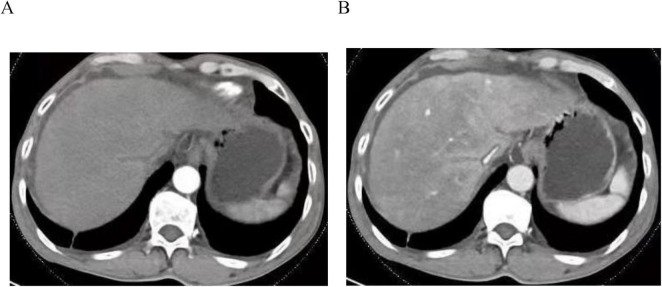
Abdominal contrast-enhanced CT scan obtained at admission. **(A)** Low enhancement during the hepatic arterial phase. **(B)** “Mosaic” enhancement in the portal venous phase, with a narrow hepatic vein lacking contrast agent filling and marked peripheral enhancement of the hepatic veins, presenting a characteristic “cloverleaf” pattern.

**FIGURE 2:**
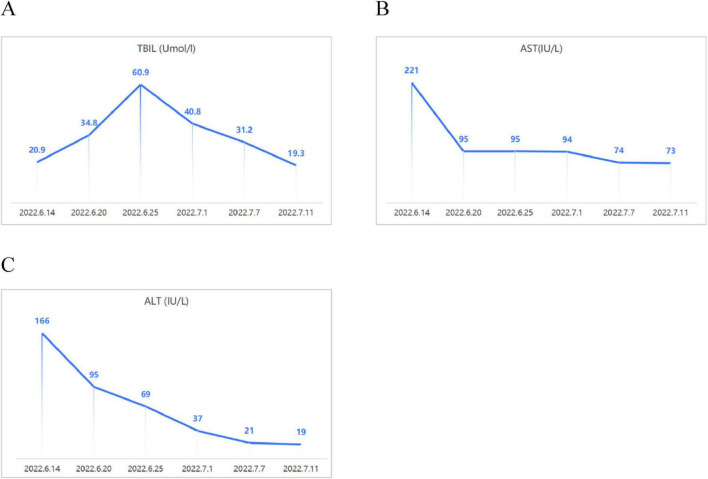
Changes in relevant indicators after treatment. **(A)** The patient’s TBIL levels initially increased, reached a peak, and subsequently declined. **(B,C)** AST and ALT levels dropped rapidly at the start of treatment and then stabilized at a lower level.

The case was sourced from Anqing Municipal Hospital. We have also ensured that all necessary ethical approvals and patient consents are appropriately addressed. A 75-year-old male patient presented with “upper abdominal distension and discomfort lasting over one month.” In May 2022, he experienced upper abdominal distension and discomfort without an identifiable cause and no associated symptoms such as acid reflux, belching, nausea, vomiting, hematemesis, melena, fever, chills, or shoulder/back pain. On June 6, 2022, gastroscopy at a local hospital identified cardia cancer and a large ulcer in the gastric angle. Pathology confirmed high-grade intraepithelial neoplasia in the cardia and chronic inflammation of the gastric angle mucosa. The patient was admitted to our department on June 13, 2022, for further evaluation and treatment. Relevant laboratory tests revealed the following results: hemoglobin 128 g/L; liver function–direct bilirubin 14.8 μmol/L, total bilirubin 20.9 μmol/L, albumin 33 g/L, prealbumin 114.2 mg/L, ALT 166 IU/L, AST 221 IU/L, ALP 221 IU/L, GGT 412 IU/L; normal coagulation function; negative HBV and HCV; elevated CA125 at 107.82 U/ml with normal CEA, CA199, and AFP levels. On June 14, contrast-enhanced CT of the chest (Figures 1A, B), abdomen, and pelvis indicated early gastric cancer, a large ulcer in the gastric angle, suspected HSOS, and ascites. Further history-taking revealed six months of Tu-San-Qi-infused (A plant rich in pyrrole alkaloids) alcohol consumption. San-Qi, a member of the Araliaceae family, is a well-known traditional Chinese medicine with various functions, including promoting blood circulation, removing blood stasis, reducing swelling, alleviating pain, and preventing cardiovascular and cerebrovascular diseases. In contrast, Tu-San-Qi, a member of the Asteraceae family, is often confused with San-Qi and contains pyrrolizidine alkaloids, which can lead to HSOS. For the past six months, Tu-San-Qi has been consumed daily as an infused liquor, although the exact dosage remains difficult to determine. The primary purpose of its use is to prevent cardiovascular and cerebrovascular diseases, and the herb was purchased by relatives from other regions. Infectious disease consultation and additional tests showed negative autoantibody profiles, a ceruloplasmin level of 45.55 mg/dL, and negative IgM antibodies for EBV, Toxoplasma, rubella virus, cytomegalovirus, and herpes simplex virus. However, total bilirubin progressively increased from 20.9 μmol/L at admission to a peak of 60.9 μmol/L before gradually normalizing. Changes in relevant indicators after admission are shown in Figure 2.

**FIGURE 3:**
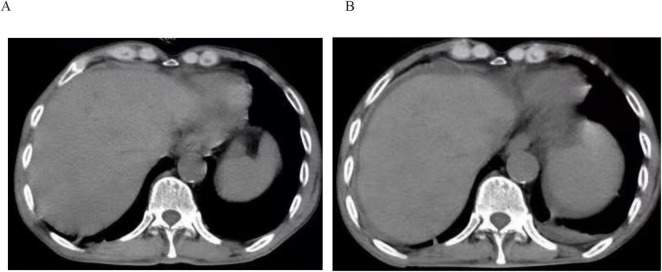
Abdominal CT images before and after radical total gastrectomy. **(A)** Abdominal CT image taken before radical total gastrectomy. **(B)** Abdominal CT image taken after radical total gastrectomy.

On July 11, follow-up abdominal and pelvic CT revealed a significant reduction in ascites (Figure 3A). On July 13, follow-up tests revealed total bilirubin of 18.8 μmol/L, ALT 18 IU/L, AST 73 IU/L, and albumin 31.3 g/L. Preoperative Child-Pugh score: A (6 points). On July 15, the patient underwent radical total gastrectomy and liver biopsy under general anesthesia. Postoperatively, the patient received continued treatments, including liver protection, albumin infusion, diuretics, and nutritional support. Low-molecular-weight heparin was added for anticoagulation 24 h postoperatively. On the first postoperative day, 1,250 ml of pale yellow ascitic fluid was drained via the abdominal drain, with approximately 1,000 ml of drainage per day over the next five days. The drainage gradually decreased after five days, remaining between 200 ml and 500 ml. On August 1 (postoperative day 17), the drainage tube was inadvertently dislodged, with 370 ml drained in the 24 h preceding dislodgement. The drainage site was sutured, and treatment was continued with albumin supplementation, diuretics, and anticoagulation. The patient exhibited no significant abdominal distension. On August 6, follow-up abdominal and pelvic CT revealed a small amount of fluid in the abdomen and pelvis (Figure 3B). The patient was discharged on August 8.

**FIGURE 4:**
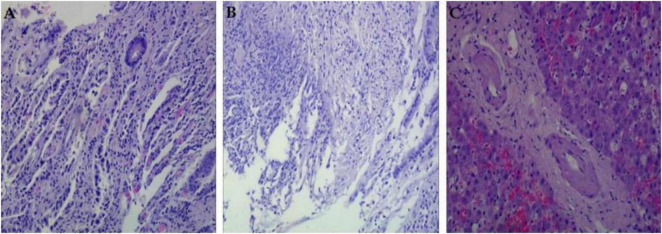
Pathological findings of the resected specimen. **(A)** High-grade intraepithelial neoplasia with carcinoma at the esophagogastric junction. **(B)** Inflammatory exudation, necrosis, and granulation tissue proliferation in the gastric antrum. **(C)** Hepatocellular edema, hemorrhage, dilation, and venular stenosis.

Postoperative pathology (Figure 4): High-grade intraepithelial neoplasia of the cardia glands with carcinoma (confined to the lamina propria), inflammatory exudative necrosis in the gastric antrum with granulation tissue proliferation consistent with ulcerative changes, a very low-risk stromal tumor in the gastric fundus, and no lymph node metastasis (0/26). Liver biopsy revealed edema, dilation, and hemorrhage of hepatic venules, consistent with hepatic sinusoidal obstruction syndrome. TNM stage: pT1aN0cM0.

### 2.1 Diagnostic criteria

The Nanjing criteria (14): the patient had a confirmed history of PA ingestion and met the following diagnostic criteria: (1) clinical manifestations, including abdominal distension, liver pain, ascites, and jaundice; (2) elevated serum total bilirubin or other liver function abnormalities; and (3) characteristic CT or MRI imaging findings. The patient was definitively diagnosed with gastric cancer complicated by HSOS and was treated with hepatoprotective therapy, albumin infusion, diuretics, nutritional support, and low-molecular-weight heparin anticoagulation.

### 2.2 Differential diagnosis

Budd-Chiari syndrome: Caused by stenosis or occlusion of the hepatic veins or inferior vena cava, it can also present as hepatomegaly and ascites. However, the caudate lobe often exhibits hyperplasia and hypertrophy, with compensatory thickening of the short hepatic veins, and enhanced imaging shows marked enhancement in the hepatic hilum with weak peripheral enhancement.

Enhanced CT or MRI in HSOS reveals characteristic features, including varying ascites, hypoperfusion during the hepatic arterial phase, “mottled” enhancement in the portal venous phase, thin hepatic veins without contrast filling, prominent peripheral enhancement of hepatic veins showing a “cloverleaf” pattern, narrowing of the retrohepatic inferior vena cava, and no significant proliferation of the caudate lobe. Budd-Chiari syndrome, resulting from stenosis or obstruction of the hepatic veins or inferior vena cava, also presents with hepatomegaly and ascites. However, the caudate lobe typically exhibits hypertrophy, and short hepatic veins become compensatorily enlarged. Enhanced imaging demonstrates marked enhancement in the hepatic hilum, with weaker peripheral enhancement.

## 3 Discussion

The diagnosis of HSOS was confirmed by characteristic imaging findings, a six-month history of pyrrolizidine alkaloid ingestion, and liver biopsy. Despite active preoperative management, total bilirubin levels peaked on day 11 and normalized nearly a month later. Early postoperative ascitic fluid output exceeded 1,000 mL, indicating severe hepatic dysfunction and a delayed response to anticoagulation. Surgical trauma, stress, anesthesia, fasting, and hypercoagulable states further impaired liver function and worsened HSOS. In gastric cancer patients with HSOS, preoperative liver function should be optimized to Child-Pugh grade A with full anticoagulation response to reduce postoperative risks. If no significant bleeding occurs within 24 h, anticoagulation should resume with high-dose albumin, diuretics, hepatoprotective agents, and nutritional support. For refractory ascites with severe hepatic decompensation, timely TIPS therapy may be necessary.

HSOS is primarily caused by toxic injury to hepatic sinusoidal endothelial cells (HSECs). In PAs-associated HSOS, this injury results from pyrrole-protein adducts (PPAs) formed by active PAs metabolites. The condition is characterized by microvascular damage, especially in acinar zone 3, where sinusoidal injury leads to hepatic venule detachment and obstruction (15). Bone marrow progenitors can replace damaged HSECs, but toxic exposure impairs this process (16). After ingestion of PAs-containing herbs or teas, water-soluble PAs salts are absorbed and transported to the liver, where cytochrome P450 enzymes convert PAs into reactive dehydropyrrolizidine alkaloids (DHPAs) and dehydroretronecine (DHR) (17). These interact with glutathione (GSH) or proteins, forming pyrrole-glutathione conjugates or PPAs, respectively. PPAs, the main driver of PAs-induced HSOS, cause F-actin depolymerization and MMP-9-mediated extracellular matrix degradation, creating gaps between HSECs (18). Blood cells and debris enter the perisinusoidal space, forming emboli that obstruct sinusoidal blood flow. Venous narrowing and impaired outflow lead to post-sinusoidal portal hypertension (4). Central lobular venule obstruction exacerbates hepatic congestion, resulting in hemorrhagic necrosis. This cascade manifests clinically as weight gain, ascites, painful hepatomegaly, and jaundice (4).

Disease diagnosis integrates medical history, clinical symptoms, laboratory tests, imaging, biomarkers, and pathology. HSOS diagnosis follows this approach, with research mainly focusing on HSCT-associated cases, diagnosed by history and the triad of weight gain, painful hepatomegaly, and jaundice (19, 20). Research on HSOS diagnostic criteria has primarily focused on hematopoietic stem cell transplantation-associated HSOS (HSCT-HSOS). The diagnosis of HSCT-HSOS relies primarily on medical history and the classic triad of weight gain, painful hepatomegaly, and jaundice. Given the similar clinical manifestations of PAs-HSOS and HSCT-HSOS, researchers have established diagnostic criteria for PAs-HSOS based on those for HSCT-HSOS. However, key differences exist between the two. HSCT-HSOS presents acutely with a short disease course (weeks), whereas PAs-HSOS has a prolonged latency period (weeks to months). Their treatment strategies also differ: HSCT-HSOS requires defibrotide therapy, whereas PAs-HSOS necessitates discontinuation of the offending agent and supportive care. Prognostically, HSCT-HSOS carries a higher risk of multiple organ failure and mortality (21, 22). Therefore, a history of PAs exposure is essential for diagnosing PAs-HSOS. However, PAs binding to serum proteins limits their diagnostic utility, and most hospitals lack testing capabilities.

Recent studies have outlined the imaging features of PAs-induced HSOS and its diagnostic value (9, 23, 24). Pathological findings remain definitive for diagnosis, and imaging techniques and liver biopsy are now key components of the Nanjing criteria proposed by the Chinese Society of Gastroenterology (7, 25). However, prospective data on its performance are lacking, requiring further research to assess its accuracy in large cohorts. Another challenge is the classification of disease staging and severity, which remains unclear due to the absence of relevant data. Imaging techniques such as ultrasound, CT, and MRI are effective for evaluating liver lesions and diagnosing liver diseases. Ultrasound, in particular, is a cost-effective tool for diagnosing PAs-induced HSOS (26). Doppler ultrasound findings include hepatomegaly, decreased portal vein flow, and hepatic vein stenosis, with characteristic features like heterogeneous arterial-phase enhancement, delayed portal vein filling, and prolonged arteriovenous transit time (27, 28). Early studies using small sample sizes identified ascites, mottled hepatic enhancement, right hepatic vein stenosis, hepatomegaly, and gallbladder wall thickening as common CT features (29). Quantitative CT analysis shows that the liver lesion-to-volume ratio correlates with clinical course and prognosis (30). MRI offers distinct diagnostic advantages without the radiation risk of CT and has proven effective in detecting oxaliplatin-induced HSOS in metastatic colorectal cancer patients (31, 32). In addition, patients with gastric cancer complicated by HSOS often develop severe hepatic decompensation. If liver function does not recover despite prolonged anticoagulation, hepatoprotective therapy, or even TIPS, the surgical risk increases substantially. Additionally, standard chemotherapy for gastric cancer may further increase the risk of HSOS. Recent studies indicate that the supernatant of *Saccharomyces boulardii* probiotic culture, a natural biological agent, exhibits anticancer effects against human gastric and colorectal cancers without significantly affecting liver function, suggesting its potential as a treatment option (33–35).

Liver biopsy and histological examination remain the gold standards for diagnosing HSOS. HSOS caused by PAs demonstrates both acute and subacute/chronic features. In the early stages, the earliest identifiable histological change is the widening of the subendothelial space between the basement membrane and the adventitia of central and sublobular veins. In acute stages, swelling, injury, and detachment of HSECs are observed in acinar zone 3 (36). Significant dilation and congestion of hepatic sinusoids occur, accompanied by centrilobular hepatocyte necrosis and the extravasation of red blood cells into the space. Thickening of the walls of small intrahepatic veins, along with lumen narrowing and occlusion, is frequently observed. In advanced stages, extracellular matrix deposition in the subendothelial space and sinusoids, along with extensive collagenization of sinusoids and small veins, constitutes characteristic histological features (22). Additionally, in subacute or chronic stages, complete loss of centrilobular hepatocytes and sinusoidal dilation are observed (26). Moreover, the uneven distribution of HSOS lesions undermines the reliability of histopathological analysis. Although transjugular liver biopsy and hepatic venous pressure gradient (HVPG) provide valuable diagnostic information, these techniques require specialized equipment and are costly, limiting their widespread adoption in many hospitals.

The management of PAs-induced HSOS remains a significant challenge, as no definitive treatment for pyrrolizidine alkaloid poisoning exists. Treatment strategies include discontinuing PAs exposure, symptomatic treatment, anticoagulation, transjugular TIPS, and liver transplantation. Individualized approaches are essential, depending on disease severity and stage. In mild cases, symptomatic treatment alone is sufficient. In severe cases, symptomatic treatment, including discontinuing PAs exposure, liver protection, and ascites management, is crucial (37). Ascites management involves restricting water and sodium intake, administering diuretics, albumin infusion, repeated paracentesis, and TIPS if necessary. Furosemide and spironolactone are the first-line diuretics, and albumin infusion is recommended for hypoalbuminemia. TIPS is indicated for refractory ascites. Patients with multi-organ failure may require hemodialysis and mechanical airway support.

Recent studies have assessed anticoagulation therapy for PAs-induced HSOS. A retrospective study from Nanjing Drum Tower Hospital found that anticoagulation therapy (low-molecular-weight heparin combined with warfarin) significantly improved remission rates compared to the non-anticoagulation group (60% vs. 27%) (27). A similar study from the First Affiliated Hospital of Zhengzhou University confirmed these results (38). Based on this, Chinese guidelines recommend initiating anticoagulation therapy early in acute or subacute HSOS patients after ruling out contraindications. Low-molecular-weight heparin is the preferred anticoagulant, administered at 100 IU/kg subcutaneously every 12 h, followed by oral warfarin with a target international normalized ratio (INR) of 2.0–3.0. However, no prospective multicenter studies have determined the efficacy of anticoagulation therapy, highlighting the need for future randomized controlled trials. Defibrotide, approved in Western countries for HSCT-related HSOS, is not approved in China, and its efficacy in PAs-induced HSOS is unclear. Liver transplantation may be considered for severe cases with liver failure, potentially improving survival. Other therapies, including ursodeoxycholic acid, antithrombin III, and recombinant human thrombomodulin, have been trialed in HSCT-related HSOS but lack evidence for PAs-induced HSOS.

In summary, for patients with gastric cancer and concurrent HSOS, surgery should be postponed until liver function has largely normalized, achieving Child-Pugh grade A and a complete response to anticoagulant therapy. This approach helps prevent postoperative refractory ascites and liver failure. If no significant bleeding occurs within 24 h postoperatively, anticoagulant therapy should be resumed, supplemented with symptomatic treatments, including albumin infusion, diuretics, hepatoprotective agents, and nutritional support. If postoperative refractory ascites develops, accompanied by severe abdominal distension and significant hepatic decompensation, timely TIPS intervention may be required.

## Data Availability

The raw data supporting the conclusions of this article will be made available by the authors, without undue reservation.
